# Psychosis in an adolescent girl: a common manifestation in Niemann-Pick Type C disease

**DOI:** 10.1186/1753-2000-8-20

**Published:** 2014-07-11

**Authors:** Sara Wouters, Linda De Meirleir, Edward Campforts, Annik Lampo

**Affiliations:** 1 Child and adolescent psychiatry, PAika - UZ Brussel, Laarbeeklaan 101, Brussels 1090, Belgium; 2 Child neurology, metabolic diseases, 2UZ Brussel, Laarbeeklaan 101, Brussels 1090, Belguim

**Keywords:** Niemann-pick type C, Psychosis, Psychiatric signs, Children, Adolescents

## Abstract

Niemann-Pick disease type C (NP-C) is a rare autosomal-recessively inherited lysosomal storage disorder. It is caused by mutations in the NPC1 (95%) or NPC2 gene. It is a progressive and highly heterogeneous disease, characterized by the presentation of visceral, neurological, and psychiatric symptoms.

Apart from the patients that die early from organic failure, most of the patients with juvenile and adolescent/adult onset of the disease, develop neurological and psychiatric symptoms. In some cases psychiatric signs, mostly psychosis, can be the first sign of the disease. A delay in diagnosis is often seen.

By describing the case of a 16-year old girl, we would like to highlight current opinion about NP-C disease and resume recent findings on the clinical presentation, diagnosis and treatment. We focus on the psychiatric signs, and most important the specific combinations that are typical for the disease.

There is no curative treatment for NP-C. Miglustat is used to modify neurological signs in NP-C.

## Background

Niemann-Pick disease type C (NP-C) is a rare autosomal-recessively inherited lysosomal storage disorder. It is caused by mutations in the NPC1 (95%) or NPC2 gene. The gene-mutations give characteristic abnormalities in the intracellular transport of endocytosed cholesterol with sequestration of unesterified cholesterol in lysosomes and late endosomes. These trafficking defects also cause accumulation of glycosphingolipids in various tissues, including the brain. The estimated incidence of diagnosis is of 1:100,000-120,000 live births [1,2].

NP-C is a neurovisceral disease, with a highly heterogeneous presentation and is characterized by progressive neurological deterioration and early death. Patients can show visceral, neurological and psychiatric manifestations which present alone or in different combinations [1-4]. NP-C disorder is chronic, but has a great variation in age of onset and disease course [2,4-7]. Niemann-Pick type C often remains undetected for many years. There is an average delay in diagnosis of 5–6 years from onset of the neurological symptoms [1,8,9]. The course towards a diagnosis even tends to be longer, when the first presentation is a psychiatric problem [10].

The clinical presentation varies with age at disease onset. It ranges between a rapidly progressive and fatal early onset form, and an adolescent/adult-onset form presenting as a chronic neurodegenerative disease [9]. We can distinguish by onset a pre-/perinatal period (≤3 months), an early infantile form (onset between 3 months of age and 2 years), a late infantile form (onset between 2 – 6 years), a juvenile form (6 – 15 years) and an adolescent/adult onset form (onset from 15 years of age) [1]. Most patients become dependent within a mean delay of 8 years [9] and the majority dies between 10 and 25 years of age [2].

In this article the authors discuss the current findings on the clinical presentation of NP-C, with focus on psychiatric manifestations. We present the case of a 16-year old girl showing severe psychotic symptomatology with paranoid delusions and visual and auditory hallucinations.

## Case presentation

A 16-year old girl, showing psychotic symptomatology (auditory and visual hallucinations, paranoid delusions) was admitted to the psychiatric clinic. Psychotic symptomatology had started abruptly. MRI and EEG were normal at this time. Her medical history showed several episodes of visceral and neurological dysfunction. The specific combination of these manifestations, the course of her disease and the age of onset, made us suspect NP-C.

We hereby present the details of our case and compare them with findings in recent literature.

Our patient’s medical history showed two unexplained episodes of splenomegaly (age 1 and age 4). A decrease of the splenic volume was seen in between episodes, a complete normalization is documented from the age of 6.

At a pre-school age, she showed a mild delay in developmental milestones. Later on she showed some mild cognitive regression signs during childhood (language problems and learning difficulties), for which she attended a specialized school and had extra speech-therapy. She was treated with methylphenidate for ‘attention deficit hyperactivity disorder’ in primary school. She had a normal social and emotional development.

Evaluation of cognitive functioning showed a slow cognitive decline since infancy. As 7-year old girl she had a normal IQ (WISC showed total IQ of 78, VIQ: 90; PIQ: 72), at the age of 13 her IQ reached a level of mental retardation (WISC-III showed total IQ of 60).

At age 11 she developed a motor regression with severe weakness. It was diagnosed as Guillain-Barré syndrome, type Miller Fisher, due to a mycoplasma pneumonia infection. CSF-analysis showed an increased protein level. After revalidation she regained partial, but good, motor capacities, she was again able to attend school.

At the age of 15 our patient started to show a slow loss of motor functioning. Ataxia and swallowing problems appeared and revalidation was intensified. Several months later, at the age of 16, she developed a severe psychotic syndrome, characterized by auditory hallucinations (hearing people talk to each other), visual hallucinations (insects running on walls, seeing unknown people) and paranoid delusions (feeling followed, feeling looked at, feeling threatened), agitation and anxiety. At administration of atypical neuroleptic drugs, she started to show dysarthria that was primarily allocated to the treatment. The neurological examination showed increased motor loss, swallowing problems and vertical supranuclear gaze palsy (VSGP).

The combination of psychotic symptoms, progressive cognitive decline, a history of splenomegaly, VSGP and loss of motor function, were suspicios for NP-C. Fibroblast filipin staining and DNA sequencing confirmed the diagnosis. Our patient appeared to be heterozygous for two mutations in the NPC1-gene. She showed one known frameshift mutation with premature stop codon (Thr124fsX4), which was clearly deleterious and one unknown missense mutation (Cys468Gly).

Since she showed major psychotic symptomatology, neuroleptics were started. Several atypical neuroleptics where used. Full remission of the psychotic sings was only seen with a high dosage (300 mg/ 2 weeks) depot form of olanzapine. Lorazepam, a psycholeptic drug, was added to decrease anxiety problems. Confirmation of the diagnoses was a very difficult period for the patient and her family. She developed a major depression that was treated with antidepressants. With this treatment we saw a remission of the psychiatric symptomatology, the neuromotor deterioration could not be stopped ever since. She is again attending specialized school in a revalidation center. She lives in her home environment.

Miglustat is the only disease specific treatment. Since miglustat is not reimbursed in Belgium in the indication of NP-C and since it is up till now not recognized as treatment for the psychotic symptoms in NP-C, our patient is not yet treated.

## Discussion

### Visceral manifestations in NP-C

Prolonged unexplained neonatal jaundice or cholestasis and isolated unexplained splenomegaly (historical and/or current) with/without hepatomegaly are the strongest visceral indicators for NP-C [6]. As in our patient, patients often show a history of neonatal jaundice or splenomegaly during infancy [2,5,9].

### The first psychiatric signs

Psychiatric signs and problems are very common in the juvenil and adult forms of NP-C. As mentioned earlier, psychiatric signs can be the first presentation of the disease and can remain isolated for many years [9].

The onset of psychiatric problems can be progressive or acute, with spontaneous remission and relapse. Children with juvenile onset (6–15 years) of NP-C often show school problems (attention difficulties, learning difficulties, writing problems) [9]. Our patient was treated for ADHD in primary school.

The leading psychiatric manifestations are cognitive decline and psychosis and they tend to increase in patients above 16 years of age [6,7]. Our patient showed a significant cognitive decline, ranging from a normal intelligence at age 7, to a level of mental retardation at age 13.

Patients show paranoid delusions, auditory or visual hallucinations, interpretative thoughts, behavioral disturbances, self-mutilation or social isolation [9]. Schizophrenia-like psychosis has been reported in up to 25% of the adult patients with NP-C [4].

Other types of psychiatric disturbances include depressive syndrome, bipolar disorders and obsessive- compulsive disorders [4], treatment-resistant psychiatric symptoms and disruptive or aggressive behavior [7]. Our patient developed in the immediate period after diagnosis a depressive syndrome.

In a recently published study [11], the prevalence of NP-C was evaluated in adult patients with psychosis and/or early-onset progressive cognitive decline with or without additional neurological or visceral signs suggestive of NP-C. 3 patients out of the 256 enrolled, were identified as NP-C positive and in 12 the diagnosis remained uncertain. The observed frequency was potentially higher than the incidence in the general population. The authors suggest an underdiagnosed pool of NP-C in this specific group of patients.

### *Neurological signs*

Gelastic cataplexia and VSGP are the strongest indicators of NP-C. Ataxia, clumsiness or frequent falls, dysarthria and/or dysphagia and dystonia are moderate indicators. Acquired and progressive spasticity is a weak indicator. Hypotonia, delayed developmental milestones, seizures and myoclonus are ancillary neurological manifestations [4,6,7].

Our patient only showed VSGP at the age of 16. We don’t know if it was missed or absent earlier in her life. We didn’t find any gelastic cataplexia in her medical history.

The wide range of neurological signs seen in NP-C can mimic other, more common, neurological and psychiatric diseases. In adults with NP-C reported in the literature, some initial diagnoses were Alzheimer’s disease, Parkinson’s disease, schizophrenia, Wilson’s disease, multiple sclerosis, Creutzfeldt–Jakob disease and Gayet– Wernicke encephalopathy [9]. In our patient, the neurological presentation was thought to be a Guillain-Barré syndrome, type Miller-Fisher.

### *Diagnosis*

Diagnosis of NP-C is difficult, especially when it presents as a pure psychiatric disorder [9]. Individual NP-C manifestations are not specific to the disease, but the combination of multiple signs and symptoms shows more diagnostic specificity for NP-C, which may help with disease detection [7,12]. Diagnosis of NP-C is made by physical and neurological assessment of the patient, biochemical tests involving filipin staining of skin fibroblasts, and genetic sequencing of the NPC1 or NPC2 gene [1,2,4]. Filipin staining and genetic analysis are recommended as the first-line diagnostic tests, to be carried out in parallel if possible in order to obtain complementary information [4].

The physical assessment is focusing on specific combinations of visceral-, neurological- and psychiatric symptomatology. Wijburg [6] developed a Suspicion Index (SI) tool, ranking specific symptoms within and across domains, including family members who have NP-C. They provide a risk prediction score to identify patients who should undergo testing for NP-C [13]. The SI provides a risk prediction score (RPS) based on NP-C manifestations within and across 3 domains (visceral, neurological, and psychiatric). It is a screening tool and is not to be used as a diagnostic tool [7]. Patients with a RPS ≥70 should be referred to an NP-C center for immediate testing, and scores from 40 to 69 indicate that further follow-up is required (and an NP-C center contacted for discussion). Scores below 40 indicate a low likelihood of NP-C [4].

Neonatal jaundice/cholestasis, splenomegaly, vertical supranuclear gaze palsy, cataplexy, and cognitive decline/dementia are strong predictors of NP-C, and also parents/siblings or cousins with NP-C [6,7]. Suspicion for NP-C should be raised when patients present clinical manifestations not only within, but more importantly across, multiple domains [7]. The SI tool maintains strong discriminatory power in patients >4 years but is not as useful for infants <4 years [7].

Our patient had a score of 222 on the SI (age 16). Until the age of 6, she didn’t show any neurological or psychiatric signs. She then started to show minor developmental delays. A calculation with the SI-score would have given a result of 126 at the age of 8. She then showed isolated splenomegaly in the medical history, delayed developmental milestones and ‘other psychiatric disorders’ (language and learning difficulties, ADHD). At the age of 11 when developing a massive motor regression with severe weakness, the SI-score would have been 147.

These findings highlight the need of a thorough medical examination and assessment of medical history, in all patients presenting with neurodevelopmental disorders or delay in development.

### *Treatment*

Patterson et al. give recommendations for diagnosis and treatment of NP-C. Since no curative treatment is available, a rigorous symptomatic treatment is advised [7]. Psychiatric symptoms in NP-C are often treatment-resistant [7]. In our patient, dyskinesia was primarily allocated to the neuroleptic treatment rather than seen as dystonia, a mistake that occurs frequently in NP-C [4] and should trigger awareness about the presence of a disorder of the basal ganglia [14].

Motor difficulties as ataxia and dysarthria, could wrongly be allocated to the side-effects of the antipsychotic medication. It is important to differentiate between drug effects and those due to the disease. Since a paradoxical effect to neuroleptic medication is often seen in these patients, one should consider aborting current treatment instead of increasing dosage. Atypical neuroleptics are proposed as the first choice treatment when psychotic symptoms occur [4].

Miglustat is currently the only disease-specific treatment [5]. Miglustat may act as a competitive inhibitor of the enzyme glucosylceramide synthase and thereby reduces the potentially neurotoxic accumulation of gangliosides GM2 and GM3, lactosylceramide and glucosylceramide [1]. Miglustat has an indication in the European Union for treatment of progressive neurological manifestations in adult and pediatric patients with NP-C, since 2009 [15].

Treatment with miglustat has shown to stabilize neurological symptoms in NP-C, but should be administered only when neurological symptomatology is present [4].

In a recently published case report, a complete remission of the psychotic symptomatology was seen in a patient with NP-C and psychosis [16]. Replication of these findings is needed before conclusions are made.

## Conclusion

NP-C is a severe neurovisceral lipid storage disease. It is characterized by a specific, but variable manifestation of visceral, neurological and psychiatric symptoms. Since it can show as a merely psychiatric illness, child and adolescent psychiatrists should take into account the potential presence of this underlying disorder.

There could be an underdiagnosis of NP-C patients in psychiatric populations [11]. Physical examination and assessment of medical history is compulsory when evaluating children with a neurodevelopmental disorder or delay.

Our patient showed a very slow loss of cognitive functioning, this in the background of one major motor syndrome and an acute psychotic decompensation. Genetic examination showed one previously undescribed missense mutation (Cys468Gly) in the NPC1-gene.

Patients presenting with any atypical psychiatric disorder or any progressive neurological syndrome including ataxia, early onset dementia or dystonia, in addition to isolated splenomegaly and/or vertical supranuclear gaze palsy (VSGP) abnormalities should be examined for possible NP-C [4,14]. The recently developed ‘Suspicion Index tool’ provides a risk prediction score to identify patients who should undergo testing for NP-C [13].

Patterson et al. [4] provide recommendations concerning diagnosis and treatment of NP-C patients.

Miglustat is the only disease specific medication. It shows its effects in the treatment of neurological manifestations, but is not curative. Early administration of miglustat could influence the trajectory of the disease.

### Consent

Written consent was obtained from the patient’s parents for publication of this Case Report. A copy of the written consent is available for review by the Editor-in-Chief of this journal.

## Competing interests

The authors declare that they have no competing interests.

## Authors’ contributions

SW did the literature research, supervised psychiatric examination and treatment of the patient, drafted the manuscript. LD examined the patient in the last phase, conducted medical testing, added significant information to the manuscript. EC and AL revised the manuscript critically for intellectual content. All authors read and approved the manuscript.
